# Alcohol Use and Selected Health Conditions of 1991 Gulf War Veterans: Survey Results, 2003-2005

**Published:** 2011-04-15

**Authors:** Steven S. Coughlin, Han K. Kang, Clare M. Mahan

**Affiliations:** Environmental Epidemiology Service (135), Office of Public Health and Environmental Hazards, Department of Veterans Affairs; Environmental Epidemiology Service, Office of Public Health and Environmental Hazards, Department of Veterans Affairs, Washington, DC; Environmental Epidemiology Service, Office of Public Health and Environmental Hazards, Department of Veterans Affairs, Washington, DC

## Abstract

**Introduction:**

A sizable literature has analyzed the frequency of alcohol consumption and patterns of drinking among veterans. However, few studies have examined patterns of alcohol use in veterans of the first Gulf War or factors associated with problem drinking in this population. We examined the frequency and patterns of alcohol use in male and female veterans who served in the 1991 Gulf War or during the same era and the relationships between alcohol use and selected health conditions.

**Methods:**

We analyzed data from a follow-up survey of health information among population-based samples of 15,000 Gulf War and 15,000 Gulf Era veterans. Data had been collected from 9,970 respondents during 2003 through 2005 via a structured questionnaire or telephone survey.

**Results:**

Posttraumatic stress disorder (PTSD), major depressive disorder (MDD), unexplained multisymptom illness (MSI), and chronic fatigue syndrome (CFS)–like illness were more frequent among veterans with problem drinking than those without problem drinking. Approximately 28% of Gulf War veterans with problem drinking had PTSD compared with 13% of Gulf War veterans without problem drinking. In multivariate analysis, problem drinking was positively associated with PTSD, MDD, unexplained MSI, and CFS–like illness after adjustment for age, sex, race/ethnicity, branch of service, rank, and Gulf status. Veterans who were problem drinkers were 2.7 times as likely to have PTSD as veterans who were not problem drinkers.

**Conclusion:**

These findings indicate that access to evidence-based treatment programs and systems of care should be provided for veterans who abuse alcohol and who have PTSD and other war-related health conditions and illnesses.

## Introduction

An increasing number of studies have examined the frequency and patterns of alcohol consumption in various veteran populations and the relationship between problem or hazardous drinking and other health conditions including posttraumatic stress disorder (PTSD) and major depressive disorder (MDD) (1-6). Studies of alcohol consumption and problem drinking conducted among veterans who served in certain theaters of operation (eg, Viet Nam, the Persian Gulf, Operation Iraqi Freedom, and Operation Enduring Freedom) may not be generalizable to other veteran populations. Patterns of alcohol consumption and problem drinking vary by sex, age, time since deployment or discharge from military service, and locality (7,8).

Studies of alcohol consumption in US veterans of the 1991 Gulf War have varied according to sample selection procedures (eg, studies of patients seen in clinical settings vs population-based research that is more likely to be representative of all Gulf War veterans) and by sample size, presence or absence of a nondeployed comparison group, calendar year of the survey or number of years since the conflict ended, approaches for data collection (eg, mail questionnaire, telephone survey, in-person interview), and other study design features (9). Variability has been found in the alcohol-related questions that were asked of Gulf War veterans (eg, not all studies have inquired about problem drinking or alcohol dependency).

The Institute of Medicine recently affirmed that evidence exists of an association between military service in the 1991 Gulf War and alcohol abuse and certain other health conditions and disorders (10). Associations also may exist between excessive alcohol use and adverse health conditions such as PTSD and MDD, which are associated with service in the Gulf War (1,11-13). Studies conducted in veteran and nonveteran populations have found that alcohol use disorders are more common among people with PTSD than among people without PTSD (14), although not all studies have shown an association between PTSD and alcohol use (15). Major depression, PTSD, and other anxiety disorders have been associated with alcohol use disorders in various veteran populations (16,17). Among male and female veterans with PTSD, alcohol abuse or dependence is one of the most common comorbid psychological conditions (14). Stressful exposures that occur during wartime deployment may contribute to heavy drinking among both men and women (18,19).

Few studies have examined associations between alcohol consumption and symptom-based illnesses, including chronic fatigue syndrome (CFS)–like illness and unexplained multisymptom illness (MSI), that have been associated with service in the 1991 Gulf War. Both CFS–like illness and MSI may occur as comorbid conditions among people with problem drinking.

We analyzed data from a follow-up health survey among population-based samples of 15,000 Gulf War veterans and 15,000 Gulf Era veterans (veterans who served during the same era but who were not deployed to the Persian Gulf). Data were collected from 9,970 respondents in 2003 through 2005 via a structured questionnaire or telephone survey. We examined the frequency and patterns of alcohol use in Gulf War and Gulf Era veterans and associations between problem drinking and selected health conditions, including PTSD and MDD.

## Methods

### Study population

The design of this study was cross-sectional. We obtained data from a follow-up survey to the 1995 National Health Survey of Gulf War Era Veterans and Their Families (20). The sampling frame consisted of the same 15,000 Gulf War veterans and 15,000 Gulf Era veterans who were selected for the 1995 survey. We sampled Gulf War veterans from 693,826 US troops who were identified by the US Department of Defense Manpower Data Center in Monterey, California, as being deployed to the Persian Gulf area during the 1991 Gulf War. We sampled Gulf Era veterans from 800,680 people who represented about one-half of all troops who were in the military between September 1990 and May 1991 but who did not serve in the Persian Gulf theaters of operations. Each branch of service (Army, Navy, Air Force, and Marine Corps) and unit component (active, reserve, and National Guard) were represented in both groups. We applied a stratified random sampling method to ensure that women and those who served in the reserve or National Guard were adequately represented (20). Approximately 20% of the sample were women.

Procedures for ascertaining the vital status and updated address of each of the sampled respondents have previously been reported (20,21). A total of 393 of the sampled veterans were deceased by the beginning of the data collection period, leaving 29,607 veterans eligible to participate in the survey. The National Health Survey of Gulf War Era Veterans and Their Families was approved by the Washington, DC, Veterans Affairs Medical Center's Human Studies Subcommittee.

### Data collection methods

Data collection began in June of 2003. We used a modified Dillman method and sent a prenotification letter to each potential participant, which was followed by questionnaire mailings conducted in 3 waves (22). The first wave consisted of mailing a 20-page structured health questionnaire to each veteran, together with an introductory letter, a consent form, and a preaddressed, stamped return envelope. The second wave mailing took place 10 weeks after the first wave mailing. The third wave mailing was conducted during a 10-week period. We then sent postcard reminders 2 weeks after each questionnaire mailing (waves 1, 2, and 3). In the second phase of data collection, we attempted telephone interviews of 2,000 veterans who had not yet responded by using computer-assisted telephone interviewing software. We completed all data collection by the end of May 2005. A total of 9,970 of the 29,607 veterans (response rate 34%) consented and participated in the postal or telephone survey. Characteristics of the respondents and nonrespondents by mode of survey have previously been reported (21).

The survey questionnaire included questions about functional status, activity limitations, health perceptions, height, weight, chronic medical conditions, PTSD and other mental disorders, and health care use. We asked the respondents whether they ever drink alcohol (including beer or wine) and, if so, they were asked about the average number of drinks they consume per week. We asked the respondents whether any of the following had happened to them more than once during the past 6 months: 1) You drank alcohol even though a doctor suggested that you stop drinking because of a problem with your health; 2) You drank alcohol, were high from alcohol, or hungover while you were working, going to school, taking care of children, or engaged in other responsibilities; 3) You missed or were late for work, school, or other activities because you were drinking or hungover; 4) You had a problem getting along with other people while you were drinking; and 5) You drove a car after having several drinks or after drinking too much.

We used the PTSD checklist (PCL) to assess symptoms of PTSD. Respondents rate PCL items on a 5-point Likert scale to indicate the degree to which they have had each of 17 PTSD symptoms during the past month (23). Possible PCL scores range from 17 to 85. In this study, we considered respondents with a PCL score of 50 or higher to have PTSD.

We used the Patient Health Questionnaire (PHQ) to assess MDD and other mental disorders. The PHQ is a brief self-report assessment of common mental disorders developed specifically for primary care (24). PHQ allows brief provisional primary care diagnoses of certain disorders, including MDD and probable alcohol abuse or dependence.

We modified the 1994 Centers for Disease Control and Prevention case definition of CFS illness (25) for use in this study because of differences in time frame (21). Therefore, we use the term "CFS–like illness" here. CFS–like illness consisted of persistent problems in the past 12 months with fatigue lasting more than 24 hours after exertion and persistent problems with a least 3 of 7 symptoms (headaches, sore throat, tender lymph nodes, muscle aches or cramps, joint aches or pain, awaken feeling tired or worn out after a full night of sleep, and difficulty concentrating or reasoning or memory loss) and none of the following medical condition exclusions: arthritis, skin cancer, any other cancer, cirrhosis of the liver, hepatitis, diabetes, other endocrine disorder, repeated seizures or convulsions or blackouts, neuralgia or neuritis, disease of the genital organs, coronary heart disease, stroke or cerebral vascular accident, tachycardia or rapid heart rate, asthma, emphysema or chronic bronchitis, and repeated bladder infections.

We defined unexplained MSI by using self-reported information about unexplained physical symptoms and illnesses (eg, fatigue, muscle or joint pain, headaches, memory problems, digestive problems, respiratory problems, skin problems) that persisted for 6 months or longer and that are not adequately explained by an established, conventional medical or mental disorder diagnosis. Such unexplained physical symptoms and illnesses, which are often not labeled, are sometimes diagnosed as CFS, fibromyalgia, irritable bowel syndrome, or multiple chemical sensitivity.

### Variables used in the analysis

Variables used in this analysis were age, sex, race/ethnicity (white, black, Hispanic, other), education (<high school; high school, general education diploma, or equivalent; some college, no degree; associate degree; bachelor's degree; or graduate degree [master's, doctorate, or professional degree]), income (<$20,000, $20,000-$34,999, $35,000-$49,999, $50,000-$74,999, $75,000-$99,999, or ≥$100,000), unit component (active, reserve, or National Guard), branch of service (Army, Navy, Air Force, or Marine Corps), rank (officer, warrant officer, or enlisted), deployment status (Gulf War or Gulf Era), CFS–like illness, unexplained MSI, PTSD, and MDD. We included categorical variables for frequency of alcohol consumption and problem drinking. We categorized respondents as nondrinkers, light drinkers (1-2 drinks per week), moderate drinkers (<3-14 drinks per week), and heavy drinkers (≥15 drinks per week on average). We defined problem drinking as an affirmative response to any of the 5 previously mentioned questions about problem drinking or hazardous drinking in the past 6 months. Exploratory analyses were conducted to examine the patterns of alcohol consumption and problem drinking among the veterans.

### Statistical analysis

For the multivariate analyses, we performed logistic regression to examine problem drinking as a predictor of the health conditions and illnesses of interest (PTSD, MDD, unexplained MSI, CFS–like illness) while controlling for all other variables included in the model (age, sex, race/ethnicity, branch of service, rank, and deployment status). We used the Hosmer-Lemeshow goodness of fit test to assess model adequacy (26). We used SAS statistical package version 9.1 (SAS Institute, Inc, Cary, North Carolina) in the analysis.

## Results

In 2005, the mean age of Gulf War veterans was 45.5 years, and the mean age of Gulf Era veterans was 47.6 years. Self-reported information from Gulf War and Gulf Era veterans indicated that alcohol consumption was similar between the 2 groups (Table 1). Approximately 30% of Gulf War and Gulf Era veterans abstained from consuming alcohol, and the percentages of Gulf War and Gulf Era veterans who were light, moderate, and heavy drinkers were almost the same. Problem drinking was attributed to 16% of Gulf War veterans and 12% of Gulf Era veterans (Table 1).

Heavy alcohol consumption and problem drinking were more frequent among Gulf War veterans who had served as enlisted personnel compared with those who had served as officers or warrant officers. Approximately 17% (898 of 5,132) of veterans who had served as enlisted personnel in the Gulf War reported problem drinking compared with 11% (94 of 872) of veterans who had been officers and 6% (5 of 86) of veterans who had been warrant officers. Heavy alcohol consumption and problem drinking were also more frequent among Gulf War veterans who had less education or lower income. For example, 17% (13 of 75) of Gulf War veterans with less than a high school education reported problem drinking compared with 11% (86 of 778) of veterans with a master's, doctorate, or professional degree.

Without adjusting for the variables, PTSD, MDD, unexplained MSI, and CFS–like illness were more frequent among veterans with problem drinking than those without problem drinking (Table 2). Approximately 28% of Gulf War veterans with problem drinking had PTSD compared with 13% of Gulf War veterans without problem drinking.

In multivariate analysis (Table 3), problem drinking was positively associated with PTSD, MDD, unexplained MSI, and CFS–like illness after adjustment for age, sex, race/ethnicity, branch of service, rank, and deployment status. In the model for PTSD, the adjusted odds ratio for problem drinking was 2.72. Lower rank and being a veteran of the Gulf War were positively associated with PTSD, MDD, unexplained MSI, and CFS–like illness. Older age, female sex, and black race were positively associated with PTSD, MDD, and unexplained MSI. Results obtained using the Hosmer-Lemeshow test indicated that all 4 logistic models fitted the data well.

## Discussion

We used cross-sectional data from a health survey of veterans that was conducted 13 years after the end of the 1991 Gulf War to examine the relationships between PTSD, MDD, and alcohol consumption. Therefore, the cases of PTSD identified in this cohort of Gulf War and Gulf Era veterans represent those that occurred as a result of war-time trauma and that persisted for an extended time, combined with PTSD cases that resulted from traumatic experiences that may have occurred after the war.

PTSD is an anxiety disorder that can occur after someone experiences a traumatic event such as a combat experience, a motor vehicle crash, or sexual assault (27). Symptoms of PTSD may include nightmares, intrusive thoughts, or other re-experiencing phenomena; the avoidance of situations that remind the person of the traumatic event; a feeling of numbness or being socially detached from family and friends; and hyperarousal (eg, feeling angry, irritable, and "on edge"; having difficulty concentrating). Hyperarousal or hypervigilance is characterized by a rapid and pronounced reaction to stressors, which may lead to a preoccupation with signs of threat and emotional distress. People with PTSD may have other challenges such as difficulties with employment, difficulties with relationships, or other health conditions (eg, depression, alcohol abuse, drug dependency) (27).

Our findings indicate that, several years after the Gulf War ended, there was a high frequency of problem drinking among Gulf War and Gulf Era veterans who have PTSD, major depression, and other illnesses and health conditions. Psychological hypotheses about the relationship between alcohol abuse and PTSD often posit that PTSD precedes the development of alcohol abuse (14). According to this hypothesis, alcohol problems may occur as a consequence of PTSD (16). Excessive alcohol consumption and alcohol dependence may result from attempts to "self-medicate" or alleviate disturbing memories or other symptoms associated with PTSD. Alternatively, shared stressors such as war-time traumas may independently lead to both PTSD and problem drinking. This latter possibility has sometimes been referred to as the "shared stressor hypothesis" (16). Because of its cross-sectional nature, our analysis does not provide evidence with which to determine which of these 2 hypotheses has more merit.

Our study, which was a national sample of Gulf War veterans, was conducted by using mail questionnaires and telephone interviews rather than face-to-face evaluations. The Iowa Gulf War Case Validation Study, conducted during 1999-2001, used face-to-face evaluations with 602 veterans sampled from a population-based survey of 4,886 military personnel and found that lifetime history of alcohol abuse or dependence was frequent among both deployed and nondeployed veterans who were depressed (68% and 52%, respectively). A study of the postwar hospitalization experience of US veterans who served in the 1991 Gulf War, which was based on medical records databases, found that alcohol dependence syndrome was the most frequent mental disorder; hospitalization rates for alcohol dependence were somewhat higher than those observed for other veterans from the same era who did not go to the Persian Gulf (standardized rate ratio = 1.19, 95% confidence interval, 1.10-1.30) (28). PTSD, major depression, and other mental disorders have previously been studied in a sample of 1,061 deployed veterans and 1,128 nondeployed veterans who participated in the National Health Survey of Gulf War Era Veterans and Their Families (3). The prevalence of depression and anxiety declined during a 10-year period among both groups but remained higher in the deployed group (3).

Our study confirms that some veterans who experience MSI or CFS–like illness also experience problem drinking as a comorbid condition. The exposures that account for increased risks of MSI and CFS–like illness among Gulf War veterans are unknown but may include environmental factors such as pyridostigmine bromide or exposure to certain pesticides (10). Many of the troops were exposed to an array of wartime and environmental exposures including psychological stress, solvents, fuels, and pesticides, pyridostigmine bromide pills given to protect troops from effects of nerve agents, smoke from oil-well fires, and prophylactic vaccines given to protect against anthrax and other infectious agents (10).

Our study has limitations. The cross-sectional nature of the analysis prevents us from making inferences about the causality of the observed associations. Another limitation is that PTSD was assessed by using the PCL screening test rather than clinical interviews; therefore, misclassification of PTSD status may have occurred. Also, information was not collected about binge drinking, which has been found to be a risk factor in other military and veteran populations (29). Furthermore, we relied on self-reported information about the frequency of alcohol use and problem drinking, which may have introduced social desirability bias; however, self-reported information about alcohol consumption has been found to be reliable and valid (30). Finally, data were not collected on illicit drug use.

In summary, the results of this survey conducted during 2003-2005 indicate that veterans of the 1991 Gulf War, particularly those who have PTSD or MDD, have a higher frequency of heavy alcohol consumption and problem drinking. These findings underscore the importance of sustained efforts to provide access to evidence-based treatment programs and systems of care for veterans who abuse alcohol and who have PTSD and other war-related health conditions and illnesses (14,27,31).

## Figures and Tables

**Table 1:** Descriptive Characteristics of Gulf War and Gulf Era Veterans Who Participated in the 2003-2005 Follow-Up Survey^a^

**Characteristic^b^**	Gulf War, No. (%) (n = 6,111)	Gulf Era, No. (%) (n = 3,859)
**Sex**		
Male	4,886 (79.9)	3,008 (78.0)
Female	1,225 (20.1)	851 (22.0)
**Race/ethnicity**		
White	4,654 (76.3)	3,131 (81.2)
Black	1,011 (16.6)	496 (12.9)
Hispanic	266 (4.3)	129 (3.4)
Other	171 (2.8)	97 (2.5)
**Branch of service**		
Air Force	745 (12.2)	534 (13.9)
Army	3,938 (64.4)	2,480 (64.3)
Marine Corps	642 (10.5)	360 (9.3)
Navy	786 (12.9)	483 (12.5)
**Unit component**		
Active	2,180 (35.7)	1,538 (39.9)
National Guard	1,774 (29.0)	1,037 (26.9)
Reserve	2,157 (35.3)	1,282 (33.2)
**Rank**		
Enlisted	5,151 (84.3)	2,943 (76.3)
Officer	874 (14.3)	831 (21.6)
Warrant officer	86 (1.4)	83 (2.1)
**Education**		
<High school	76 (1.3)	46 (1.2)
High school, GED, or equivalent	1,148 (19.0)	538 (14.2)
Some college, no degree	2,021 (33.5)	1,062 (28.0)
Associate degree	803 (13.3)	515 (13.5)
Bachelor's degree	1,205 (20.0)	897 (23.6)
Master's, doctorate, or professional degree	778 (12.9)	739 (19.5)
**Income, $**		
<20,000	558 (9.5)	243 (6.5)
20,000-34,999	974 (16.5)	535 (14.3)
35,000-49,999	1,271 (21.5)	707 (18.9)
50,000-74,999	1,578 (26.7)	978 (26.1)
75,000-99,999	783 (13.3)	578 (15.4)
≥100,000	739 (12.5)	705 (18.8)
**Average number of drinks per week**		
0 (nondrinker)	1,687 (28.4)	1,102 (29.3)
1-2 (light drinker)	1,877 (31.6)	1,231 (32.8)
3-8 (moderate drinker)	1,389 (23.4)	880 (23.4)
9-14 (moderate drinker)	501 (8.4)	291 (7.7)
≥15 (heavy drinker)	479 (8.1)	255 (6.8)
**Problem drinking**		
Yes	997 (16.4)	461 (12.0)
No	5,093 (83.6)	3,384 (88.0)

Abbreviation: GED, general education diploma.

a Data are self-reported. Some categories may not sum to total because of missing data.

b In 2005, mean age of Gulf War veterans (n = 6,102) was 45.5 years, and mean age of Gulf Era veterans (n = 3,857) was 47.6 years.

**Table 2:** Unadjusted Percentages of Self-Reported Health Outcomes and Medical Conditions Among Gulf War and Gulf Era Veterans With and Without Problem Drinking^a^

Health Outcomes	Gulf War Veterans (n = 6,111), Problem Drinking		Gulf Era Veterans (n = 3,859), Problem Drinking	

	Yes, No. (%)	No, No. (%)	Yes, No. (%)	No, No. (%)
**Functional impairment^b^**				
Yes	391 (39.7)	1,517 (30.0)	108 (23.6)	524 (15.6)
No	595 (60.3)	3,544 (70.0)	350 (76.4)	2,845 (84.5)
**Limitation of activities^c^**				
Yes	296 (30.0)	1,451 (28.8)	112 (24.5)	622 (18.5)
No	692 (70.0)	3,594 (71.2)	345 (75.5)	2,744 (81.5)
**Has a doctor ever told you that you have . . .**				
**Cirrhosis of the liver**				
Yes	96 (9.9)	402 (8.1)	31 (6.9)	209 (6.3)
No	876 (90.1)	4,576 (91.9)	418 (93.1)	3,127 (93.7)
**Hepatitis**				
Yes	129 (13.3)	518 (10.4)	44 (9.8)	295 (8.9)
No	844 (86.7)	4,467 (89.6)	405 (90.2)	3,040 (91.2)
**Gastritis**				
Yes	297 (30.3)	1,338 (26.8)	98 (21.7)	585 (17.6)
No	682 (69.7)	3,660 (73.2)	354 (78.3)	2,747 (82.4)
**Seizures, convulsions, or blackouts**				
Yes	113 (11.6)	488 (9.8)	37 (8.2)	225 (6.8)
No	860 (88.4)	4,483 (90.2)	412 (91.8)	3,110 (93.3)
**Hypertension**				
Yes	328 (33.4)	1,476 (29.4)	153 (33.5)	918 (27.5)
No	654 (66.6)	3,537 (70.6)	304 (66.5)	2,424 (72.5)
**Tachycardia**				
Yes	161 (16.6)	684 (13.7)	51 (11.4)	329 (9.9)
No	809 (83.4)	4,298 (86.3)	398 (88.6)	3,009 (90.1)
**Posttraumatic stress disorder**				
Yes	278 (28.0)	643 (12.7)	53 (11.5)	123 (3.6)
No	716 (72.0)	4,440 (87.4)	408 (88.5)	3,254 (96.4)
**Major depressive disorder**				
Yes	245 (24.6)	654 (12.9)	60 (13.0)	164 (4.9)
No	750 (75.4)	4,429 (87.1)	400 (87.0)	3,213 (95.1)
**Unexplained multisymptom illness**				
Yes	433 (44.7)	1,740 (34.9)	78 (17.1)	367 (10.9)
No	536 (55.3)	3,251 (65.1)	379 (82.9)	2,989 (89.1)
**Chronic fatigue syndrome–like illness (past 12 months)**				
Yes	127 (12.7)	445 (8.7)	36 (7.8)	96 (2.8)
No	870 (87.3)	4,648 (91.3)	425 (92.2)	3,288 (97.2)

a The percentages shown in this table represent the percentage of Gulf War or Gulf Era veterans with or without problem drinking according to whether they had the health condition of interest. Numbers vary slightly because of missing data.

b Positive response to the question, "Thinking back over the past 2 weeks, did you stay in bed or at home all or part of any day because you did not feel well or as a result of illness or injury?"

c Positive response to the question, "Are you limited in your employment or the kind of work you can do around the house because of any impairment or health problem?"

**Table 3:** Adjusted Odds Ratios From Logistic Regression Modeling of Selected Health Outcomes in Gulf War and Gulf Era Veterans

Covariate	Model 1: PTSD (n = 9,900), AOR^a^ (95% CI)	Model 2: MDD (n = 9,900), AOR^a^ (95% CI)	Model 3: Unexplained MSI (n = 9,758), AOR^a^ (95% CI)	Model 4: CFS–Like Illness (n = 9,920), AOR^a^ (95% CI)
**Age, y**	1.00^b^ (1.00-1.02)	1.01^b^ (1.00-1.02)	1.01^c^ (1.00-1.02)	0.95^c^ (0.94-0.96)
**Sex**				
Male	1 [Reference]	1 [Reference]	1 [Reference]	1 [Reference]
Female	1.21^b^ (1.03-1.43)	1.33^c^ (1.14-1.56)	1.51^c^ (1.34-1.70)	0.84 (0.68-1.03)
**Race**				
Black	1 [Reference]	1 [Reference]	1 [Reference]	1 [Reference]
White	0.54 (0.46-0.63)	0.64 (0.55-0.75)	0.70 (0.61-0.79)	0.91 (0.74-1.13)
Other	0.80^c^ (0.62-1.04)	0.83^c^ (0.64-1.08)	0.95^c^ (0.77-1.17)	0.94 (0.66-1.33)
**Branch of service**				
Air Force	1 [Reference]	1 [Reference]	1 [Reference]	1 [Reference]
Army	2.75 (2.08-3.64)	2.01 (1.57-2.57)	1.62 (1.39-1.89)	1.34 (1.01-1.78)
Marines	1.69 (1.18-2.42)	1.40 (1.01-1.94)	1.20 (0.96-1.49)	1.30 (0.91-1.85)
Navy	1.76^c^ (1.25-2.47)	1.49^c^ (1.10-2.02)	0.92^c^ (0.75-1.13)	0.97^b^ (0.67-1.40)
**Rank**				
Enlisted	1 [Reference]	1 [Reference]	1 [Reference]	1 [Reference]
Officer/warrant officer	0.32^c^ (0.25-0.42)	0.42^c^ (0.33-0.52)	0.62^c^ (0.54-0.72)	0.53^c^ (0.39-0.72)
**Deployment status**				
Gulf Era	1 [Reference]	1 [Reference]	1 [Reference]	1 [Reference]
Gulf War	3.40^c^ (2.87-4.03)	2.60^c^ (2.23-3.04)	4.33^c^ (3.86-4.85)	2.52^c^ (2.07-3.07)
**Problem drinking**				
No	1 [Reference]	1 [Reference]	1 [Reference]	1 [Reference]
Yes	2.72^c^ (2.33-3.16)	2.32^c^ (1.99-2.70)	1.56^c^ (1.37-1.77)	1.48^c^ (1.22-1.78)

Abbreviations: PTSD, posttraumatic stress disorder; AOR, adjusted odds ratio; CI, confidence interval; MDD, major depressive disorder; MSI, multisymptom illness; CFS, chronic fatigue syndrome.

a Adjusted for age, sex, race/ethnicity, branch of service, rank, and deployment status.

b *P* < .05 calculated from Wald *χ* ^2^ test.

c *P* < .001 calculated from Wald *χ* ^2^ test.
